# The Impact of Continuous and Partial Reinforcement on the Acquisition and Generalization of Human-Conditioned Fear

**DOI:** 10.3390/bs14080630

**Published:** 2024-07-24

**Authors:** Yidan Song, Shaochen Zhao, Muxin Rong, Ying Liu, Yu Gao, Wei Chen, Donghuan Zhang, Xifu Zheng

**Affiliations:** 1 Key Laboratory of Brain, Cognition and Education Sciences, Ministry of Education, South China Normal University, Guangzhou 510631, China; 2 School of Psychology, Center for Studies of Psychological Application, Guangdong Key Laboratory of Mental Health and Cognitive Science, South China Normal University, Guangzhou 510631, China; 3 Research Center for Guangdong-HongKong-Marcao Policing Model Innovation, China People’s Police University, Guangzhou 510663, China

**Keywords:** partial reinforcement, continuous reinforcement, fear generalization, skin conductance

## Abstract

Fear over-generalization as a core symptom of anxiety disorders is manifested by fear responses even to safe stimuli that are very dissimilar to the original dangerous stimulus. The present study investigated the effects of two separate conditioned stimuli–unconditioned stimuli (CS–US) pairing procedures on fear acquisition and generalization using a perceptual discrimination fear-conditioning paradigm, with US expectancy ratings and skin conductance response (SCR) as indicators. One group accepted continuous followed by partial CS–US pairings (C–P group); the other group accepted partial followed by continuous CS–US pairings (P–C group). It was found that compared to the P–C group, the C–P group showed stronger perceptual discrimination of CS+ and CS− in the fear acquisition and showed weaker SCRs and stronger extinction of US expectancy in the generalization. These findings emphasize that CS–US pairings significantly influence fear acquisition and generalization and suggest that continuous-following partial CS–US pairings promote individual discrimination of threat and safety signals and inhibit the generalization of conditioned fear. The results of this study have implications for clinical interventions for patients experiencing negative events.

## 1. Introduction

When an individual acquires fear, the fear response is generally not limited to the initial fear stimulus but spreads to other stimuli that are similar to the initial stimulus. This phenomenon is known as fear generalization [1]. Appropriate fear generalization has positive evolutionary implications, allowing individuals to respond quickly to potentially dangerous stimuli and situations, thus preparing for “fight or flight” behaviors and avoiding injury [2]. However, fear overgeneralization is considered to be one of the central characteristics of psychological problems such as anxiety disorders [3]. Fear overgeneralization has been found in clinical patients with generalized anxiety disorder, panic disorder, and social anxiety disorder. Individuals with anxiety disorders tend to perceive uncertain information in the environment as threatening and are more sensitive to potential threats. It is difficult for them to correctly distinguish between safety signals and danger signals, and they exhibit excessive worry, anxiety, and even avoidance behaviors by rapidly extending their fear or anxiety to harmless environmental cues [3,4,5,6].

Discrimination fear conditioning has become a popular paradigm for studying the learning and generalization that may mediate anxiety-related disorders. In this paradigm, a previously neutral conditioned stimulus (CS+) is repeatedly paired with an aversive unconditioned stimulus (US), while another conditioned stimulus (CS−) is presented without the US [7]. Individuals are considered to have acquired the relationship between CS and US when they demonstrate a stronger conditioned response (CR) to CS+ than to CS−. The response CR occurs not only for specific CS+ but also for neutral stimuli similar to CS+ (generalization stimuli [GS]), which is a fear generalization. Fear generalization is influenced not only by subjective factors such as personal knowledge and experience (such as category concepts) and cognitive beliefs but also by objective perceptual characteristics of the stimulus itself [8]. Of these, environmental complexity based on multiple factors (e.g., reinforcement rate) is known to affect the strength and memory of conditioned responses [9,10]. A continuous reinforcement procedure means that CS+ is consistently followed by US (i.e., 100% reinforcement rate), whereas a partial reinforcement procedure means that CS+ is only followed by US in a subset of the acquired trials (i.e., <100% reinforcement rate). Fear-generalization studies have found a fear-enhancing effect using partial reinforcement/pairing procedures that increase fear responses to GS [10]. However, there is additional evidence indicating that such procedures may interfere with initial learning. During acquisition, the percentage of CRs expressed is proportional to the CS–US pairing rate [11]. Continuous CS–US pairing procedures result in rapid CR acquisition, while partial CS–US pairing procedures serve to slow down the extinction process [12,13]. Thus, it is necessary to conduct additional research in order to gain a deeper understanding of the impact of these conditioned processes on fear acquisition and generalization and inform the design of future studies in this field.

Currently, there has been more attention paid to the effect of partial CS–US pairing on fear acquisition in Pavlovian conditioning. In contrast to continuous pairings, partial pairings may lead to slower CR acquisition and weaker CR expression [11,14,15]. Previous studies have shown that compared to continuous-pairing procedures, partial-pairing procedures reduce the strength of CS–US associations, and continuous and partial reinforcement may involve different patterns of learning in fear conditioning [16,17]. The fMRI study confirmed that the patterns of brain activity in fear conditioning were also not consistent in continuous and partial reinforcement. Compared with a 100% reinforcement rate, the insula and dorsolateral prefrontal cortex (dlPFC) were more strongly activated in the group using a 50% reinforcement rate. The insula and dlPFC may mediate processes associated with the uncertainty of US presentation [18]. Some studies have used a combination of continuous and partial reinforcement procedures. In a mixed procedure, continuous reinforcement trials are followed by partial reinforcement trials (or vice versa) during a single acquisition phase [19]. Perry and Moore’s eyeblink conditioning study comparing continuous, partial, and mixed partial-then-continuous pairing procedures found that continuous pairs showed the strongest conditioned response during fear acquisition, followed by mixed partial-continuous pairs, and partial pairs [20]. Grady et al. also compared four groups of different types of CS–US pairings and found that the mixed partial-then-continuous procedures showed the strongest conditioned response in fear acquisition and maintained conditioned response better during extinction [21]. However, similar results were not found for the mixed continuous-then-partial procedures, implying that the order of presentation of continuous and partial reinforcement in the mixed-pairing procedure should also be attended to.

Most studies of CS–US reinforcement/pairing effects on fear generalization used partial reinforcement, although some studies used continuous reinforcement [22,23,24]. Using the partial-pairing procedure in these studies was intended to retard extinction, with the expectation that partial CS–US pairing would enhance resistance to extinction and prolong the duration required to assess the fear-generalization phase. Previous studies have also corroborated the notion of the swift extinction of conditioned responses following continuous CS–US pairing in fear conditioning [25,26,27,28]. Zhao et al. used 50%, 75%, and 100% reinforcement rates for fear-acquisition training, respectively, and found that during the generalization phase, the partial-reinforcement (50% and 75%) groups showed stronger generalization on US expectancy compared to continuous reinforcement (100%) [10]. That is, due to the uncertainty of the threat stimulus, the partial-reinforcement procedure elicited more anxiety and thus increased the fear response to the GS. Although Pavlovian conditioning studies have explored CS–US pairing rates, there remains a significant gap in understanding mixed CS–US pairing procedures, particularly for fear generalization. Due to the importance of these different methodological procedures for understanding the mechanisms of anxiety disorders and fear responses, it is necessary to explore whether partial and continuous CS–US pairings influence conditioned fear acquisition and generalization in humans. We investigated the effects of varying CS–US reinforcement/pairing on the acquisition and generalization of conditioned skin conductance response (SCR) during discrimination fear conditioning while monitoring US expectancy to investigate the effects of continuous and partial pairing processes on subjective expectancy of threat.

## 2. Methods and Materials

### 2.1. Participants

The sample size was based on power analyses carried out with G–power [29]. In order to detect medium effect sizes (Cohen’s *f* = 0.25) with 80% power for Stimulus type × Generalization phase × Group ANOVAs, a minimum of twenty participants were required. Based on this, we recruited much more participants. Fifty-two healthy participants were randomly selected into the mixed continuous-then-partial (C–P) group or partial-then-continuous (P–C) group, with twenty-six participants in each group. Inclusion criteria for participants were normal vision or corrected vision, no hearing impairment, no history of physical or mental illness, and not having participated in similar research in our laboratory within the last three months. All participants completed the State–Trait Anxiety Inventory and the Intolerance of Uncertainty Scale (IUS) before the experiment. The study was approved by the human research ethics committee for non-clinical faculties at the School of Psychology. All participants provided written informed consent in accordance with the Declaration of Helsinki and received a certain reward after the experiment.

Due to instrument failure during the experiment, data from two participants were excluded. The analysis took place with data from 50 participants (33 women and 17 men; mean *SD* age = 19.73 ± 1.95 years; range, 18–26 years). There was no significant difference in age between the P–C group (16 women and 9 men) and the C–P group (17 women and 8 men), *F*(1, 48) = 0.75, *p* = 0.383; no significant difference in trait anxiety, *F*(1, 48) = 0.10, *p* = 0.755, and state anxiety, *F*(1, 48) = 0.02, *p* = 0.879; and no significant difference between inhibitory anxiety, *F*(1, 48) = 3.00, *p* = 0.90, and prospective anxiety, *F*(1, 48) = 1.19, *p* = 0.281, on the IUS.

### 2.2. Stimuli and Measures

#### 2.2.1. Stimuli 

**Figure 1:**
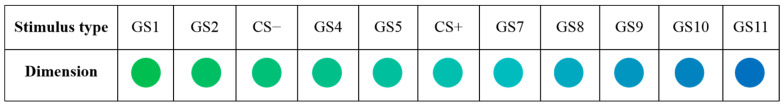
Conditioned Stimuli (CS) and Generalization Stimuli (GS). The dimensional orientation (from GS1 to GS11) was balanced (green-blue or blue-green) between participants. GS6 as CS+ and GS3 (or GS9) as CS−.

Two practice stimuli, a white circular L1 with a diameter of 200 pixels and a white square L2 with each side measuring 200 pixels, were utilized. In the formal experiment, 11 circular stimuli (200 pixels in diameter) showed hue variations along the green-blue dimension (Figure 1) [30]. Stimuli were generated by varying the hue, while saturation and brightness were kept at 100% and 75%, respectively. The maximum and minimum values of hue were 145 and 195, respectively, which varied equally between stimuli along the dimensions. The hue dimension directions (from GS1 to GS11) were counterbalanced (green to blue or blue to green). The intermediate stimulus was chosen as CS+ (GS6) among the 11 stimuli. Instead of choosing the endpoint stimulus as the safe stimulus, as in previous studies [31,32,33], we chose the stimulus with two distance points from the endpoint stimulus as the CS−. The CS− (GS3 or GS9) was greener or bluer than CS+. Participants were counterbalanced using either GS3 or GS9 as CS− for training.

The Digitimer DS2A constant current stimulator (Hertfordshire, UK) was attached to participants’ right wrists with disposable electrodes during the experiment. The stimulator generated a 500 ms shock as unconditioned stimulation (US). The intensity of the shock was individualized for each participant. Prior to the formal experiment, participants rated the shock tolerance on a scale of 1–9, choosing a rating of 8 (i.e., “uncomfortable but still tolerable”) as the US for that participant in the formal experiment. Specifically, the average intensity of US administration was 43.52 V (range: 30–62 V) for the C–P group and 45.36 V (range: 27–62 V) for the P–C group.

#### 2.2.2. Online Expectancy Rating of US

US expectancy ratings were measured online during stimulus presentation. A scoring scale was displayed at the bottom of the screen, ranging from “Definitely no shock” (1) to “Not sure” (5) to “Definitely a shock” (9). Participants were scored by pressing keys.

#### 2.2.3. Skin Conductance Response 

Skin conductance response (SCR) data were collected using a Biopac MP150 system (BioPac Systems Inc., Goleta, CA, USA) that connected electrodes to the second and third fingertips of the participant’s left hand and sampled at 1000 Hz. We used AcqKnowledge 4.2 (Biopac Systems Inc, CA, USA) to process SCR waveforms offline. Based on the SCR response before the stimulus and the SCR rise time after stimulus onset (i.e., the time required for the SCR to peak), we selected the average skin conductance level 1 s prior to stimulus as the baseline and the highest value within 8 s post-stimulus onset as the response maxima and calculated the difference between the maximum and the minimum values [23,34]. SCRs were considered to be associated with stimulus presentation if the through-to-peak response was occurring from 1 s pre-stimulus onset to 8 s post-stimulus onset and was greater than 0.02 µs [28,35]. If these criteria were not met, the SCR score was replaced with 0 [36]. Then, for each participant, these amplitudes were range-corrected, taking the highest value induced by the US as the maximum range [37,38]. All SCRs were square-root transformed to normalize distributions [39].

### 2.3. Experimental Procedure 

The experiment was run on a computer using EPRIME 2.0. Before the experiment, the skin conductance and shock electrodes were attached to each participant, and the intensity of the shock was determined for each individual. The experiment consisted of habituation, acquisition, and fear generalization phases. Details of the trials of the conditioned stimulus and generalized stimulus at each stage are shown in Table 1.

#### 2.3.1. Habituation

The practice stimuli L1 and L2 were presented three times, respectively. The purpose of the exercise was to familiarize participants with the rules of operation and the experimental interface. No shocks were administered and no data were collected following stimuli.

#### 2.3.2. Acquisition

There were two blocks in the acquisition phase (block 1 as the early acquisition and block 2 as the late acquisition), CS+ and CS−, with four trials in each block, respectively. A pseudo-randomized order of presentation of the CS was used by ensuring that the same stimulus type did not occur more than twice in a row. Participants were asked to use a numeric keypad to rate the likelihood of a subsequent US appearance during circle presentation from 1 (definitely no shock) to 9 (definitely a shock) in text displayed simultaneously beneath the stimulus. In the P–C group, the total reinforcement rate of CS+ was 75%, in which block 1 was administered by 50% shock and block 2 by 100% shock. In the C–P group, the total reinforcement rate of CS+ was also 75%, in which block 1 was administered by 100% shock and in block 2 by 50% shock; the CS− were never reinforced. During acquisition, the first CS+ trial was always paired with shock; the nature of the first stimulus (CS+ vs. CS−) was counterbalanced across participants. 

#### 2.3.3. Generalization

There were six blocks in the generalization phase, each containing 13 trials, of which there were two presentations of CS+ (one of which was followed by the shock), two presentations of CS−, and one presentation of each of the nine GS. Participants were required to make a subjective assessment of the likelihood of subsequent US appearances when the circle was presented based on the patterns learned during the fear acquisition phase. The GS and CS− were never followed by the shock.

The exact experimental procedure was as follows: participants were informed that circles would appear on the screen, followed by the possibility of an electrical stimulus. The experiment started with a fixation point “+” in the center of the screen for 500 ms, followed by 8000 ms of CS or GS, during which the likelihood of a US was judged. Then, a US or blank screen with 500 ms was presented immediately after the CS or GS offset [37,40]. Trial intervals varied from 13 to 17 s, with a mean time of 15 s. Following fear conditioning, participants had a 5 min break. To ensure that each participant engaged in the same task-irrelevant activity during this short break, a segment of a neutral video of a train traveling through British Columbia was shown [41]. The same experimental procedure was used for the fear acquisition phase and generalization phase.

### 2.4. Statistical Analysis

During the acquisition phase, US expectancy ratings and SCR data were binned into trial blocks for early acquisition (4 CS+ and 4 CS− trials) and late acquisition (4 CS+ and 4 CS− trials) phases before statistical analyses. The SCR and US expectancy were assessed by repeated-measures analysis of variance (ANOVA). A 2 (Stimulus type) × 2 (Acquisition phase) × 2 (Group) ANOVA was completed with stimulus type (CS+ and CS−) and phase (early acquisition, late acquisition) as the within-group factor and group (P–C group and C–P group) as the between-groups factor. For the generalization phase, an 11 (Stimulus type) × 6 (Generalization phase) × 2 (Group) ANOVA was completed with stimulus type (GS1, GS2, CS−, GS4, GS5, CS+, GS7, GS8, GS9, GS10, GS11) and phase (Gene1, Gene2, Gene3, Gene4, Gene5, Gene6) as the within-group factor and group (P–C group and C–P group) as the between-groups factor. The Greenhouse–Geisser correction was used to correct the degree of freedom when Mauchly’s test indicated that the sphericity assumption was violated. Bonferroni correction was further applied to adjust *p*-values for all pairwise comparisons. We adopted a significance level of 0.05, and we reported *η_p_*^2^ as the estimate of effect size.

## 3. Results

### 3.1. Acquisition

#### 3.1.1. US Expectancy

**Figure 2:**
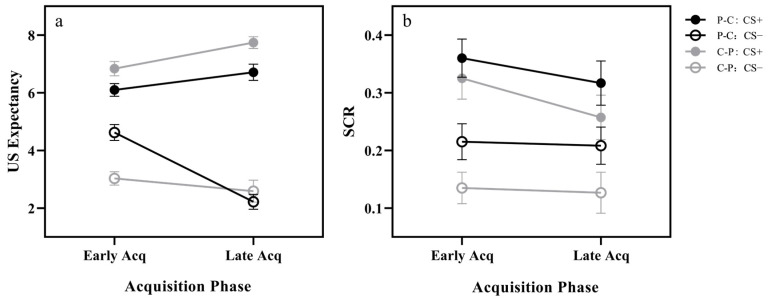
US expectancy and skin conductance response. (**a**) Unconditioned stimulus (US) expectancy and (**b**) skin conductance response (SCR) during early and late acquisition (Acq). P–C = partial followed by continuous CS–US pairings; C–P = continuous followed by partial CS–US pairings. Error bars indicate the standard error of the mean.

Repeated-measures ANOVA revealed significant main effects for stimulus type, *F*(1, 48) = 207.68, *p* < 0.001, *η_p_*^2^ = 0.812, and acquisition phase, *F*(1, 48) = 6.92, *p* = 0.011, *η_p_*^2^ = 0.126. There was no significance for group *F*(1, 48) = 0.44, *p* = 0.508. Furthermore, stimulus type × acquisition phase, *F*(1, 48) = 63.72, *p* < 0.001, *η_p_*^2^ = 0.570, and stimulus type × group, *F*(1, 48) = 8.33, *p* = 0.006, *η_p_*^2^ = 0.148, and acquisition phase × group, *F*(1, 48) = 19.80, *p* < 0.001, *η_p_*^2^ = 0.292, as well as stimulus type × acquisition phase × group, *F*(1, 48) = 9.40, *p* = 0.004, *η_p_*^2^ = 0.164, interactions were observed. Simple effect analyses revealed that the expectancy of CS+ was significantly greater than CS− in both groups in the early and late acquisition phases (*p*s < 0.001). For CS+, the C–P group had significantly greater expectancy than the P–C group during early and late acquisition phases (*p*s < 0.05); For CS−, the P–C group had a greater expectancy than the C–P group during the early acquisition phases (*p* < 0.001); there was no difference in expectancy between the two groups during the late acquisition phases (*p* = 0.426). To further explore the differences in fear acquisition between the two groups, we assessed the difference between CS+ and CS− expectancy (CS+ minus CS−) by a 2 (acquisition phase) × 2 (group) repeated measures ANOVA. We found a significant main effect of the acquisition phase, *F*(1, 48) = 63.723, *p* < 0.001, *η_p_*^2^ = 0.570, and the expectancy difference in the late phase was significantly greater than in the early phase. The group main effect was significant, *F*(1, 48) = 8.329, *p* = 0.006, *η_p_*^2^ = 0.148, and the P–C group had a significantly less expectancy difference than the C–P group. Acquisition phase × group interaction was significant, *F*(1, 48) = 9.392, *p* = 0.004, *η_p_*^2^ = 0.164. Further analyses revealed that the expectancy difference in the late phase was greater than the early phase in both the P–C and C–P groups (*p*s < 0.001). In the early phase, the expectancy difference between the P–C group was less than the C–P group (*p* < 0.001), and in the late phase, there was no significant difference between the two groups (*p* = 0.286). This supports that the C–P group showed stronger perceptual discrimination between CS+ and CS− in fear acquisition. In sum, although both groups successfully acquired fear, they differed in early and late phases. Specifically, the C–P group showed a stronger response to the fear signal (CS+) and acquired the safety signal (CS−) faster than the P–C group (see Figure 2a).

#### 3.1.2. Skin Conductance Response

Repeated-measures ANOVA of SCR data revealed significant main effects for stimulus type, *F*(1, 48) = 68.68, *p* < 0.001, *η_p_*^2^ = 0.589, and acquisition phase, *F*(1, 48) = 4.30, *p* = 0.044, *η_p_*^2^ = 0.082. During the acquisition phase, the SCRs of CS+ were significantly higher than those of CS−, but the responses decreased over time (see Figure 2b). There was no significance of group, *F*(1, 48) = 2.63, *p* = 0.111. No significant stimulus type × acquisition phase, *F*(1, 48) = 2.25, *p* = 0.140; stimulus type × group, *F*(1, 48) = 0.94, *p* = 0.337; acquisition phase × group, *F*(1, 48) =0.18, *p* = 0.671; stimulus type × acquisition phase × group, *F*(1, 48) = 0.18, *p* = 0.671, interactions were found.

### 3.2. Generalization

#### 3.2.1. US Expectancy

**Figure 3:**
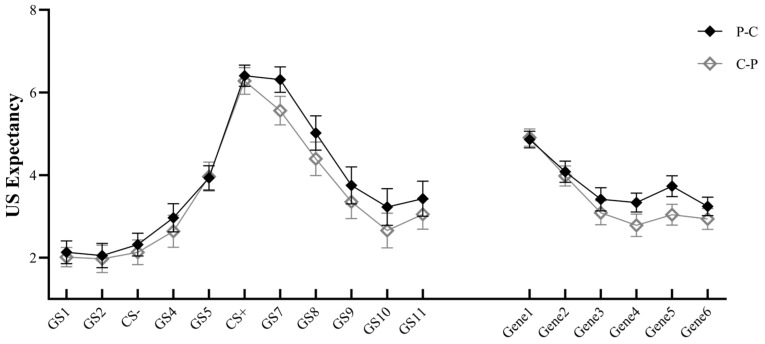
Unconditioned stimulus (US) expectancy of P–C and C–P groups under different stimulus types and generalization phases (Note: GS1–GS11 refer to different stimulus types in the generalization phase; Gene1–6 refer to 6 blocks in the generalization phase).

Repeated-measures ANOVA revealed a significant main effect for stimulus type, *F*(10, 480) = 53.14, *p* < 0.001, *η_p_*^2^ = 0.525, indicating that the closer to CS+, the higher the expectancy. The main effect was significant for generalization phase, *F*(5, 240) = 66.120, *p* < 0.001, *η_p_*^2^ = 0.579, but not for group, *F*(1, 48) = 1.049, *p* = 0.311. Stimulus type × generalization phase interaction was significant, *F*(50, 2400) = 6.601, *p* < 0.001, *η_p_*^2^ = 0.121; generalization phase × group interaction was marginally significant, *F*(5, 240) = 2.499, *p* = 0.053, *η_p_*^2^ = 0.049. Further analyses revealed that the US expectancy became smaller in both the PC and CP groups over time, showing an extinction effect (see Figure 3). To further explore the variation in generalization intensity over time between the two groups, we conducted a paired *t*-test for the difference in US expectancy at early generalization [(Gene1 + Gene2 + Gene3)/3] and late generalization [(Gene4 + Gene5 + Gene6)/3]. The P–C group (*M* = 0.682, *SD* = 0.639) was significantly smaller than the C–P group (*M* = 1.071, *SD* = 0.741), *t*(24) = −2.132, *p* = 0.043, *d* = 0.426, indicating that the C–P group had a more obvious decrease in US expectancy. The stimulus type × group interaction was not significant, *F*(10, 480) = 0.376, *p* = 0.823; the stimulus type × generalization phase × group interaction was not significant, *F*(50, 2400) = 0.799, *p* = 0.700. 

#### 3.2.2. Skin Conductance Response

**Figure 4:**
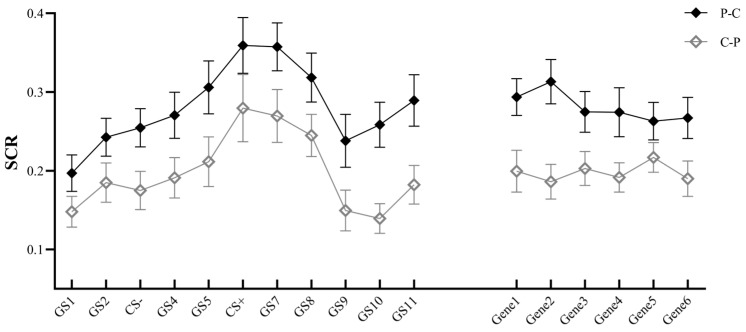
Skin conductance response (SCR) of P–C and C–P groups under different stimulus types and generalization phases (Note: GS1–GS11 refer to different stimulus types in the generalization phase; Gene1–6 refer to 6 blocks in the generalization phase).

Repeated-measures ANOVA revealed a significant main effect for stimulus type, *F*(10, 480) = 9.290, *p* < 0.001, *η_p_*^2^ = 0.162, indicating that as the stimulus was closer to CS+, SCR was higher and was easier to generalize. There was a significant main effect of group, *F*(1, 48) = 9.187, *p* = 0.004, *η_p_*^2^ = 0.161, with the P–C group having a stronger SCR than the C–P group. The generalization phase main effect was not significant, *F*(5, 240) = 0.491, *p* = 0.729. There was no significant interaction of stimulus type × group, *F*(10, 480) = 0.392, *p* = 0.842, and no significant interaction of generalization phase × group, *F*(5, 240) = 1.399, *p* = 0.239. The stimulus type × generalization phase interaction was significant, *F*(50, 2400) = 3.087, *p* < 0.001, *η_p_*^2^ = 0.060; the stimulus type × generalization phase × group interaction was significant, *F*(50, 2400) = 1.480, *p* = 0.041, *η_p_*^2^ = 0.030 (see Figure 4).

Further analyses revealed that on Gene1, for GS2, CS−, GS4, GS8, GS10, and GS11, the SCR was significantly higher in the P–C group than in the C–P group (*p*s < 0.05). For GS4, GS5, GS7, and GS10 on Gene2, the SCR was significantly higher in the P–C group than in the C–P group (*p*s < 0.05). For GS9 and GS11 on Gene3, the SCR was significantly or marginally significantly higher in the P–C group than in the C–P group (*p*s < 0.055). For GS5 on Gene4, the SCR was higher in the P–C group than in the C–P group (*p* = 0.002). Meanwhile, in the P–C group, the SCR for GS5, CS+, GS7, and GS10 was higher than CS− on Gene2 (*p*s < 0.05), and for CS+ and GS8 was significantly or marginally significantly higher than CS− on Gene4 (*p*s < 0.073); the SCR for GS5, CS+, GS7, and GS8 was higher than CS− on Gene5 (*p*s < 0.05). In the C–P group, the SCR for CS+ and GS7 on Gene1 was higher than that for CS− (*p*s < 0.05), and for CS+ and GS8 on Gene4 was significantly or marginally significantly higher than that for CS− (*p*s < 0.059); the SCR for GS4, GS5, CS+, and GS7 on Gene5 was significantly greater than CS− (*p*s < 0.05).

## 4. Discussion

The difference between continuous and partial reinforcement is key to explaining the potentially complex mechanisms of fear generalization. However, until now, no empirical studies have investigated the potential impact of the CS–US pairings on fear generalization in healthy adults. We combined the discriminative conditioning fear paradigm to explore whether partial and continuous CS–US pairings affect individual fear acquisition and generalization. We found that individuals were successful in acquiring conditioned fear regardless of whether they underwent the mixed continuous-then-partial reinforcement (C–P) or partial-then-continuous reinforcement (P–C) procedures. Additionally, the C–P group exhibited enhanced perceptual discrimination between threat and safety signals. Moreover, the C–P group exhibited weaker SCRs and stronger extinction of US expectancy compared to the P–C group. The findings of this study suggest that the C–P procedure leads to better threat discrimination and inhibits fear generalization. 

### 4.1. CS–US Pairing Affects the Discrimination of Conditioned Fear Responses 

We observed no significant difference in the acquisition of conditioned fear between the two groups, as measured by both US expectancy and skin conductance response (SCR). However, the CS–US pairing procedure did affect fear discrimination, resulting in differential performance on US expectancy at early and late acquisition. For the Pavlovian fear conditioning model in a laboratory setting, CS+ indicates a dangerous or fearful stimulus, whereas CS− represents a safety signal. Our findings reveal that the C–P group exhibited an enhanced fear response to the CS+ and an earlier acquisition of safety signal (CS−) compared to the P–C group. In general, physiological conditioned responses (e.g., SCR) are thought to reflect implicit fear conditioning, whereas explicitly expressing knowledge of programmed experimental contingencies or verbal reports of increased fear, anxiety, or expectancy are thought to reflect explicit fear conditioning, requiring higher-order cognitive processes [42,43]; all of these responses are characterized as reflecting learning and may have some overlap [43,44]. Based on this, we propose that the C–P group showed stronger perceptual discrimination, and it is cautiously hypothesized that the CS–US pairing procedure affects fear acquisition more at the explicit level.

Previous research has found that individuals are able to adaptively adjust their behavior based on the level of uncertainty in their environment [45]. Specifically, when the probability of target appearance fluctuates, individuals exhibit an increased rate of learning, enabling them to rapidly adapt to high levels of uncertainty during interactions. Conversely, when the probability of target presentation is relatively stable, the rate of learning decreases [46]. Moreover, individuals are able to adjust their learning in response to changes in target accuracy during interactions, thereby updating their subjective judgments accurately [47]. In this study, for the C–P group, the rate of CS–US pairing transitioned from high stability (100%) in early acquisition to instability (50%) in late acquisition, resulting in a gradual increase in the individuals’ rate of learning. Conversely, the US presentation of the P–C group gradually stabilized from early acquisition (50%) to late acquisition (100%), leading to a gradual decrease in individual learning rates. Dickinson argued that allocating significant cognitive resources to cues with already known outcomes is unnecessary [48]. Instead, focusing attention on cues with unknown predictability facilitates rapid learning of their true significance. Overall, the C–P group was better able to discriminate between CS+ and CS−, implying a better recognition of the difference between safety and threat signals. Although similar results are not observed at the implicit physiological level, from an evolutionary perspective, subjectively “exaggerated” expectancy of harmful outcomes that may occur in a given situation may be intended to alert peers for support or to warn peers to avoid potential dangers that may be present [49]. Individuals need to mobilize resources to cope with the danger signals while inhibiting the fearful response to safety signals. This type of discrimination learning helps individuals to effectively respond to threatening stimuli to ensure better adaptation to the environment [22].

### 4.2. Mixed Partial-then-Continuous Reinforcement Enhances Fear Generalization

There is evidence suggesting that the CS–US reinforcement/pairing also influences fear generalization. Over time, both groups exhibited a decrease in US expectancy. Despite variations in continuous and partial pairing procedures, individuals demonstrated fear extinction, particularly in the C–P group, which exhibited more robust extinction. This suggests that the P–C reinforcement procedure may result in a relatively well-maintained conditioned US acquisition during the generalization phase, aligning with previous findings indicating that the P–C procedure can enhance resistance to extinction [19,21].

We also found that participants in both groups exhibited a generalization of fear response towards stimuli that approximated CS+ and had significantly increased SCRs in the P–C group as compared to the C–P group. The observed differences in the generalization phase may be attributed to the CS–US reinforcement procedure employed during the fear acquisition. Specifically, during the acquisition phase, participants in the C–P group who received a 100% continuous reinforcement followed by a 50% partial-reinforcement schedule reached a state of anticipatory uncertainty. Upon entering the generalization stage, there was also ambiguity regarding the relationship between the CS and US and GS and US [50]. This uncertainty may have masked the transition to generalization [51], making it challenging for participants in the C–P group to perceive any changes in the environment or the beginning of a new stage. In contrast, for the P–C group, who received a 50% partial reinforcement followed by a 100% continuous reinforcement schedule, the continued reinforcement of US in the late phase led to a state of anticipatory certainty. Consequently, the increase in response to GS during generalization may be attributed to the fact that participants in the P–C group recognized that the stimulus contingency had changed and were able to transition to the new phase with relative clarity.

Interestingly, the generalization results reveal a significantly greater intensity of fear response in the P–C group compared to the C–P group, as indicated by the SCRs, while no significant difference was observed in the US expectancy between the two groups. Given this weak separation of subjective expectancy and SCR, Lissek [52] proposed that fear generalization involves two mechanisms. On the one hand, there is the processing pathway for a conditioned danger cue (CS+), which primarily involves the thalamus, amygdala, subcortical and brainstem structures (e.g., the lateral hypothalamus, central grey, and bed nucleus of the stria terminalis). The intensity of activation in these brain regions increases as GS and CS+ come progressively closer [22], and this fear activation is mainly reflected in physiological indices [53]. On the other hand, there is a discriminative processing pathway for a conditioned safety cue (CS−), which mainly involves the hippocampus, ventromedial prefrontal cortex, and anterior insula. The closer the GS is to the CS−, the stronger the activation of the associated brain regions. This process is mainly reflected in subjective rating indicators [53,54]. 

The dual-process model holds that the subjective ratings and physiological responses of fear conditioning are the result of the interaction of two independent mechanisms [43], allowing for a separation between independent explicit and implicit processes. Lesion and neuroimaging studies provide evidence for the dual-process model. Studies of brain lesions in human subjects have found that when the amygdala is damaged, the patient is able to express CS–US contingent awareness but does not express conditional SCR. In contrast, the patient with hippocampal damage is able to obtain conditional SCR but has no awareness of CS–US contingencies [55]. The patient with damage to the hippocampus and amygdala is unable to express conditional SCR and fails to acquire the awareness of stimulus contingencies [55]. Evidence for a dual process of fear conditioning has also been found in studies of healthy human participants [56,57,58]. Thus, implicit fear-conditioned responses can occur in the absence of explicit knowledge. In the present study, we proposed that the P–C reinforcement procedure may affect the discriminative processing pathway for conditioned safety cues during the generalization phase, with difficulties in inhibiting fear responses to the safety signal (CS−), resulting in the P–C group maintaining a high level of physiological alertness but not reporting high US expectancy subjectively. It also indicates that the heightened fear response observed in the P–C group was not caused by a concomitant increase in US expectancy, in part supporting the dual-process model.

### 4.3. Limitations of the Study

The current study has some limitations. We argue that CS–US pairings modulate the uncertainty state of individuals during the acquisition and generalization phases. However, there were no specific indicators to measure uncertainty. Therefore, the effect of uncertainty on fear generalization in this study should be interpreted cautiously. To avoid this limitation in future studies, subjective assessments of participants could be included at the end of the experiment. Participants could be asked a cluster of inter-linked questions, such as the following:

1. Do you think there is a relationship between the graphs presented in the experiment and the electric shocks?
2. What was the relationship?
3. How certain are you about this relationship?

Another limitation of the study is that the participants recruited for this study were physically and mentally healthy (as far as we know) rather than patients with anxiety disorders. Therefore, whether the results of this study apply to clinical patients is an issue that needs further investigation.

Although patients with clinical anxiety were not recruited for this study, the findings may have some implications for the treatment of anxiety-related disorders. Exposure therapy is a core component of cognitive-behavioral therapy; it is typically used in the treatment of anxiety disorders [59], and it is the most commonly used behavioral therapy for the treatment of neuroses such as phobias and obsessive-compulsive disorders [60]. Exposure therapy repeatedly exposes and habituates the individual to an environment that ensures safety and triggers traumatic memories. During the exposure process, the fear and pain elicited by the individual’s traumatic memories decrease, which changes the fear schema and decreases the generalization of fear [61]. We found that a mixed continuous-then-partial reinforcement procedure in fear acquisition can effectively inhibit the fear generalization to other similar stimuli and have better extinction effects (a procedure that may also be instructive for clinical interventions for patients with anxiety disorders). This suggests that during clinical exposure therapy, individuals should be trained in fear acquisition through mixed continuous-then-partial procedures to better discriminate between threat signals and safety signals, which can also be effective in reducing fear overgeneralization and improving the effectiveness of exposure therapy.

## 5. Conclusions

The present study innovatively investigated whether CS–US pairing can influence fear acquisition and generalization. We observed that compared to the P–C procedure, the C–P procedure showed stronger perceptual discrimination of threat and safety signals during fear acquisition and led to weaker fear responses during the generalization phase. These findings highlight the potential of CS–US reinforcement/pairing modifications in clinical interventions for emotional disorders, particularly fear and anxiety disorders.

## Figures and Tables

**Table 1:** Trials of conditioned stimulus and generalized stimulus at each stage.

Group	Habituation	Acquisition		Generalization
		Block1	Block2	
P–C group	3(L1), 3(L2)	4(CS+) (2US) 4CS−	4(CS+) (4US) 4CS−	6(GS1), 6(GS2), 12(CS−), 6(GS4), 6(GS5), 12(CS+), 6(GS7), 6(GS8), 6(GS9), 6(GS10), 6(GS11)
C–P group	3(L1), 3(L2)	4(CS+) (4US) 4CS−	4(CS+) (2US) 4CS−	6(GS1), 6(GS2), 12(CS−), 6(GS4), 6(GS5), 12(CS+), 6(GS7), 6(GS8), 6(GS9), 6(GS10), 6(GS11)

Note: the numbers in this table are the number of stimulus presentations.

## Data Availability

The data of this study are available from the corresponding author upon reasonable request.
